# Mechanically resilient, alumina-reinforced carbon nanotube arrays for in-plane shock absorption in micromechanical devices

**DOI:** 10.1038/s41378-023-00539-7

**Published:** 2023-06-08

**Authors:** Eunhwan Jo, Hojoon Lee, Jae-Ik Lee, Jongbaeg Kim

**Affiliations:** 1 School of Mechanical Engineering, Yonsei University, 50 Yonsei-ro, Seodaemun-gu, Seoul, 03722 Republic of Korea; 2 Department of Neurosurgery, Massachusetts General Hospital, Harvard Medical School, 25 Shattuck St, Boston, MA 02115 USA

**Keywords:** Carbon nanotubes and fullerenes, Electrical and electronic engineering, Carbon nanotubes and fullerenes

## Abstract

Microelectromechanical systems (MEMS) are of considerable interest due to their compact size and low power consumption when used in modern electronics. MEMS devices intrinsically incorporate three-dimensional (3D) microstructures for their intended operations; however, these microstructures are easily broken by mechanical shocks accompanying high-magnitude transient acceleration, inducing device malfunction. Although various structural designs and materials have been proposed to overcome this limit, developing a shock absorber for easy integration into existing MEMS structures that effectively dissipates impact energy remains challenging. Here, a vertically aligned 3D nanocomposite based on ceramic-reinforced carbon nanotube (CNT) arrays is presented for in-plane shock-absorbing and energy dissipation around MEMS devices. This geometrically aligned composite consists of regionally-selective integrated CNT arrays and a subsequent atomically thick alumina layer coating, which serve as structural and reinforcing materials, respectively. The nanocomposite is integrated with the microstructure through a batch-fabrication process and remarkably improves the in-plane shock reliability of a designed movable structure over a wide acceleration range (0–12,000*g*). In addition, the enhanced shock reliability through the nanocomposite was experimentally verified through comparison with various control devices.

## Introduction

With rapid advances in the Internet of Things (IoT), electric vehicles, and mobile technologies, there has been an increasing need for miniaturized electronics, with sizes and power consumption less than those of current devices^1^. Microelectromechanical systems (MEMS) are of great interest as essential components of such electronics for use in wireless communication, environment monitoring and inertial sensing^2–4^. Unlike solid-state devices such as semiconductor-based sensors and transistors, MEMS devices contain microstructures in which mechanical stress is inevitably induced during functional performance. Certain commercially available MEMS devices, including accelerometers, gyroscopes, optical mirrors, and radio frequency (RF) modules, include movable microstructures suspended on microbeams that are susceptible to damage via mechanical stress^5^.

Mechanical shock, a transient mechanical excitation accompanying a high-magnitude acceleration in an extremely short time, induces mechanical stress in a microbeam due to the abrupt force applied to the movable structure. Mechanical stress often causes malfunction and failure of MEMS devices owing to crack propagation or fracture in the microbeam, where stress is applied over the yield stress of the structural material^6^. The high-magnitude acceleration induced by the shock easily exceeds the critical value of the yield stress in typical applications upon exposure to a high-shock environment. For example, when portable devices are accidentally dropped (from a sufficient height) by a customer, the acceleration value can reach up to 5000*g* based on the hardness of the surface on which they land^7^. In addition, MEMS devices for automotive applications require high reliability with an acceleration of more than 20,000*g*, as may be induced in a sudden traffic accident^8^. Furthermore, device reliability should be guaranteed even at an acceleration of up to approximately 100,000*g* to realize the use of MEMS devices in harsh-environment machinery for military, aerospace, and oil and gas drilling industries^9,10^.

In terms of structural design, simply increasing the stiffness of the beam is ineffective because it degrades device performance, specifically parameters including sensitivity, operating range, and resolution^11^. A straightforward strategy that has been adopted to protect a microstructure is to add a structure that limits excessive displacement, namely, a hard stop^12^. Integrating the hard stop in MEMS suppresses the bending stress exceeding the yield stress at the susceptible position, such as the anchor part of the beam. However, when acceleration is applied to the movable structure, the impact at the hard stop causes a secondary shock, leading to additional problems, including undesired oscillation and debris generation due to the mechanical contact with the bump stop under high acceleration^13^. Moreover, the secondary shock induces additional stress on the beam, which can cause fractures at different positions, such as the supporting part of the movable structure. Therefore, alleviating the impact force between the movable structure and the hard stop is imperative to protect the movable structure and improve the reliability of the device. When shock with specific kinetic energy is applied, the impact force can be reduced by increasing the contact time or decreasing the coefficient of restitution (COR), as determined by structural and material characteristics. Accordingly, several researchers have introduced structural modifications of the hard stop to increase the contact time. They reduced the magnitude of the impact force and improved the shock reliability of microdevices by utilizing deformable microstructures such as compliant nonlinear springs^14^, flexible stops^15^, latching stops^16,17^, and cascade beams^18^. In terms of energy dissipation, some researchers have proposed various materials with low COR as contact materials, that is, shock absorbers^19^. Typically, used Si and SiO_2_ for MEMS devices have high CORs of 0.7 and 0.96, respectively^19^; however, soft materials, including ductile metals and soft polymers, have low CORs from 0.22 to 0.36^20^. Thus, researchers have improved the shock reliability of their devices by introducing these soft materials as shock absorbers in various forms, including thin films, glass beads, and solder balls^14,20,21^.

Moreover, three-dimensional (3D) nanoscale lattices have recently attracted great interest as structural materials for mechanical energy dissipation^22–24^. Their periodically patterned 3D structure results in outstanding mechanical resilience and high damping capability suitable for a shock-energy absorber; however, large-scalable and cost-effective fabrication methods are still lacking owing to their complex and serial processes. Therefore, it is essential to develop mass-producible and batch-processable manufacturing processes that can be integrated into microstructures for practical use in electronics with high shock reliability. Herein, we present a vertically aligned 3D nanocomposite based on ceramic-reinforced carbon nanotube (CNT) arrays for the in-plane shock absorption and energy dissipation materials of MEMS. This geometrically nanostructured composite consists of synthesized region-selective CNT arrays and an alumina (Al_2_O_3_) layer coated with an atomic thickness, serving as structural and reinforcing materials, respectively. The nanocomposite, integrated with the microstructure through batch-fabrication processes, remarkably improved the in-plane shock reliability of a proof mass, which is a designed movable structure, over a wide acceleration range (0–12,000*g*). When abrupt acceleration is applied, the mechanical deformability of the aligned CNT array, which as a cushioning structure, increases the contact time^25^. At the same time, the thin layer-coated Al_2_O_3_ reinforces the mechanical resilience of the composite by suppressing the internal adhesion force between the individual nanocomposite strands. We experimentally verified that the nanocomposite provides an improved shock absorption capability by comparison with various control devices, including a hard stop, a spring stop, and a CNT-based stop without ceramic layer reinforcement, as reported in the literature. In addition, after applying excessive acceleration loads, we investigated the change in the structural characteristics of both the nanocomposite and the microstructure to identify their failure mechanism.

## Results

### Design and fabrication

**Fig. 1:**
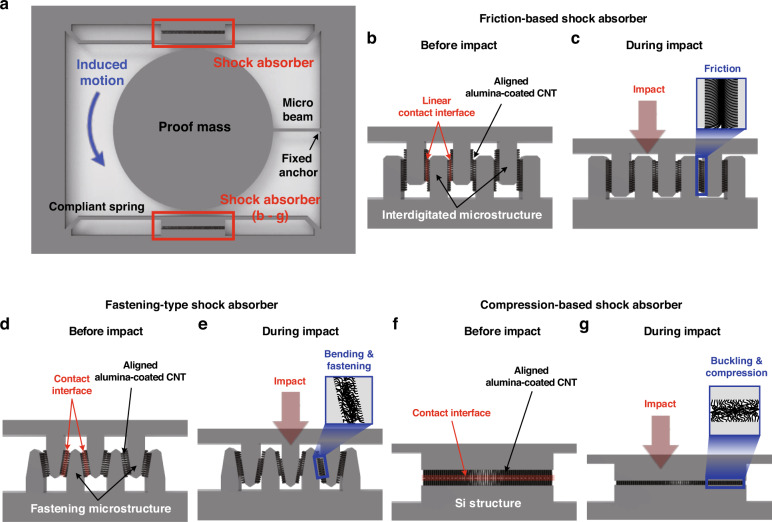
Shock absorber based on alumina (Al2O3)-reinforced carbon nanotube (CNT) arrays. **a** A schematic illustration of the nanocomposite-based shock absorbers with a proof mass suspended on a microbeam. Illustrations of the shock absorbing mechanism by using **b**, **c** friction-based, **d**, **e** fastening-type, and **f**, **g** compression-based shock absorbers, respectively. The nanocomposite arrays are directly synthesized in the sidewall of the confined microstructures and form the linear contact interface

Figure 1a shows a schematic illustration of the nanocomposite-based shock absorber with a proof mass suspended on a microbeam. The shock absorber consists of a compliant spring, fixed anchor, and a nanocomposite material that fills the gaps between them. Note that the beam supporting the proof mass is referred to as a microbeam, and the beam colliding with the proof mass is referred to as a spring. We considered three structural concepts utilizing nanocomposites to improve the shock reliability of movable structures, which are referred to as friction-based, fastening-type, and compression-based shock absorbers, respectively, as reported in our previous studies^26,27^. First, we exploit the frictional contact at the interface between the nanocomposite arrays to dissipate the mechanical shock energy by Coulomb damping. Figures 1b and 1c show a schematic diagram of a friction-based shock absorber and its mechanism, respectively. As depicted in Fig. 1b, the nanocomposite is directly integrated into interdigitated microstructures to form parallel linear contact interfaces. When frictional contact occurs at the interface, the shock energy is dissipated through frictional damping, as illustrated in Fig. 1c. The compliant spring is then restored at the position where the static frictional force is made equal to the restoring force of the spring. The second concept is a fastening-type shock absorber that simultaneously induces the bending and compression of the nanocomposite due to frictional force while reducing the gap between interdigitated structures during impact loading. Figure 1d, e shows the schematic diagram and mechanism of the fastening-type shock absorber with the nanocomposite. When a shock is applied, the gap closing induces compressive deformation in the nanocomposite by the normal load while also causing bending of the individual composite strands by the shear force due to the frictional force at the interface. This mechanical behavior of nanocomposites leads to both shock energy dissipation and increasing contact time because of the bending and compression of the nanocomposite, respectively, as shown in Fig. 1e. Finally, the compression-based shock absorber is considered the third concept of the shock absorber, as shown in Fig. 1f, g. When a collision occurs between the proof mass and the compliant spring, the high aspect ratio of the aligned structure enables compressive deformation in the nanocomposite^28^. As a result, the shock energy was dissipated during the compressive deformation, which decreased the maximum value of acceleration applied to the proof mass as the contact time increased. Then, the compressed nanocomposite regains its original state owing to reinforcement with Al_2_O_3_ after the position of the proof mass is restored.

### Characteristics of shock absorbers with nanocomposites

**Fig. 2:**
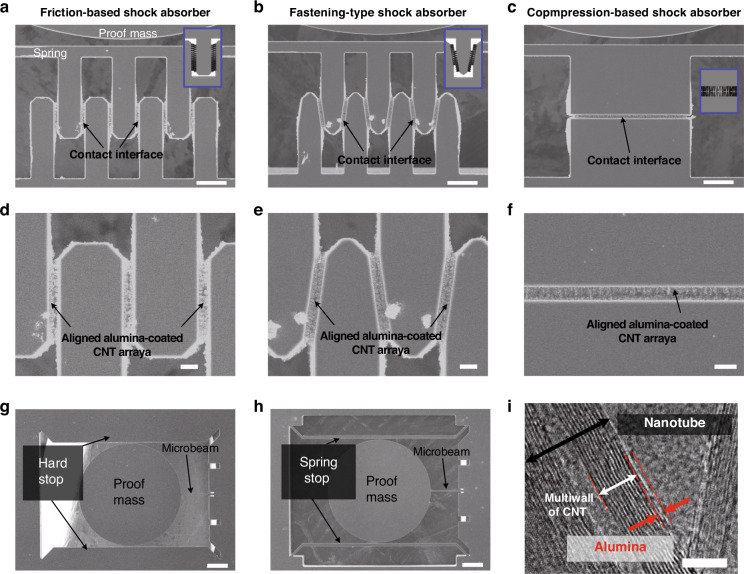
Morphological characteristics of the shock absorbers and nanocomposites. Scanning electron microscope (SEM) images of **a** friction-based, **b** fastening-type, and **c** compression-based shock absorbers with Al_2_O_3_-coated CNT arrays. The scale bars are 50 and 10 μm. **d**, **e** SEM images of a hard stop and a spring stop as a control device, respectively. The scale bar is 200 μm. **f** A transmission electron microscopy (TEM) image of the nanocomposite with the Al_2_O_3_ layer indicating the uniform coating of the individual CNTs. The scale bar is 5 nm

To confirm the successful integration of the nanocomposite in the fabricated microstructure, we investigated the morphological characteristics of the shock absorbers using scanning electron microscopy (SEM). We describe the specific fabrication process in the “Experimental Section” and Fig. S1. Figure 2a–c shows the SEM images of the proposed shock absorbers with Al_2_O_3_-coated CNT arrays. The enlarged SEM images in Fig. 2a–c and Fig. S2 show that the aligned Al_2_O_3_–carbon nanotube arrays were successfully synthesized in the designated sidewalls, forming linear contact interfaces in the confined microstructures^29^. As shown in the SEM images of Fig. 2a–c and Fig. S3, we confirmed that the conformal coating of the Al_2_O_3_ layer with a thickness of 2 nm did not cause a structural change in the aligned composite arrays.

To investigate the effect of introducing the nanocomposites, we prepared specimens with a hard stop and a spring stop as a control device. Figure 2d, e shows SEM images of a hard stop and a spring stop, adopting the structure design in the previously reported literature^14^. We also monitored individual composite strands to determine the uniformity of the Al_2_O_3_ coating. Figure 2f shows a transmission electron microscopy (TEM) image of the composite, confirming that Al_2_O_3_ uniformly coated all the surfaces of individual CNTs, which is consistent with previously reported findings in the literature verifying that the Al_2_O_3_ layer with a target thickness of 2 nm or more conformably covers CNT arrays through TEM analysis^28,30^.

## Discussion

### Shock reliability test of shock absorbers

Crucially, we experimentally measured the shock reliability of various shock absorbers over a wide acceleration range (0–12,000*g*). We prepared 56 specimens, 7 per each design, including 3 types of composite-based absorbers, 3 types of bare CNT-based absorbers, spring stops, and hard stops. The specimen chips (9 mm × 9 mm) consisted of shock absorbers with nanocomposites, a spring stop, or a hard stop as control devices and were mounted on a vertically falling carriage with guide rails. Figure S4 and Table S1 show the details of the design parameters and dimensions of the tested specimens. Then, the carriage was dropped on an aluminum-based hard substrate while controlling the drop height (see Fig. S5 for the experimental setup). The acceleration was measured using a commercially available piezoelectric accelerometer (350D02, PCB Piezotronics) that was mechanically fixed to the carriage through a tapped hole with a screw on the accelerometer. The acceleration data were extracted with the highest amplitude value of the shock-induced oscillation response within a sinusoidal half period of approximately 50 μs, as shown in Fig. S6.

**Fig. 3:**
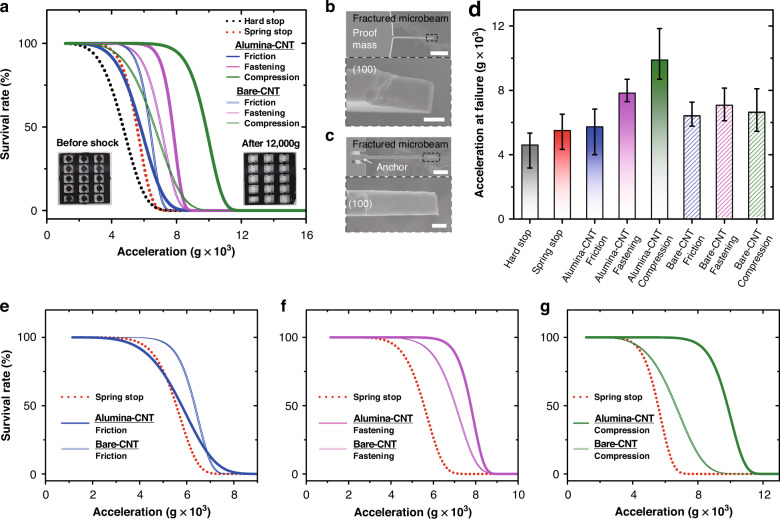
Shock-survival characteristics of the shock absorbers with the nanocomposites. **a** The surv ival rate curves of all specimens (56 specimens) with respect to the applied acceleration. The insets of **a** are representative optical images of a specimen before and after the shock test. The curves are fitted with a Weibull distribution to predict the failure rate of the devices as a function of shock exposure. **b**, **c** SEM images of the cross-sectional area of the fractured microbeam after the shock test. The scale bars in **b** and **c** are 100 and 50 μm, respectively. The scale bars in the enlarged images **b**, **c** are 5 μm. **d** The mean values and standard errors of the fracture acceleration. **e**–**g** Weibull distribution-fitted survival rate curves indicating the difference in the survival rate of the specimen with the nanocomposite, the bare CNT, and the spring stop in the case of **e** friction-based, **f** fastening-type, and **g** compression-based shock absorbers, respectively

Figure 3a shows the survival rate curves of all specimens with respect to the applied acceleration. The curves were fitted with a Weibull distribution to predict the failure rate of the devices as a function of shock exposure (see Fig. S7 and Table S2 for the specific acceleration values and the fitted parameters of the Weibull distribution). Among the parameters of the Weibull distribution, α is the acceleration at which 36.79% of the devices survive. Additionally, *β* is the Weibull modulus representing the slope of the curve. Specifically, when *β* is larger than 1, the failure rate increases with respect to an input parameter, an acceleration in our work. Additionally, the prediction of the failure becomes more accurate, and the error decreases as the value of *β* increases. The survival rate data indicate that the shock absorbers with the nanocomposite exhibit high shock-absorbing capability compared to the Si-based spring stop and the hard stop. Additionally, it was observed that the survival rate of the shock absorbers with the nanocomposite was higher than that with bare CNTs at a high acceleration of up to 12,000*g*. The fracture occurred in the microbeam suspending the proof mass in all specimens aftershock with an acceleration over 12,000*g* was applied, as shown in the inset of Fig. 3a. To investigate the fracture mechanism of the microbeams supporting the proof mass, we investigated the broken specimens after the shock survival test. In the specimens with the nanocomposite, the majority of the fracture occurred at the middle position of the microbeam, as shown in the SEM images of Fig. 3b, c. During the collision, the concentrated stress at the tip is seemingly reduced by the deformable nanocomposite. These deformable composites provide shock energy dissipation capability with increased contact time compared with those of the control devices. Moreover, both ends of the beam are fixed due to the contact between the proof mass and the shock absorber with the nanocomposites. Then, the shock-induced stress is concentrated in the middle of the beam. Thus, when an excessive acceleration of over 12,000*g* was loaded, the shock-induced stress in the middle of the beam was higher than the critical yield stress, leading to fracture in the middle of the beam. Furthermore, we observed the cross-sectional area of the broken beam. In contrast, in the case of two control devices (a hard stop and a spring stop), fractures occurred at the tip of the microbeam where the proof mass was supported. When collision occurred between the proof mass and the Si structure, it induced an impact force concentrated at the tip of the microbeam. These observed failure mechanisms of the control devices are consistent with previously reported studies^14^.

Figure 3d shows the mean values and standard errors of the fracture acceleration data. Among the devices with nanocomposites integrated into various structures, the compression-based shock absorber was observed to withstand the highest shock acceleration. Both the bare CNT array and alumina-reinforced composite exhibit mechanical deformability due to the high aspect ratio^28,31^. This mechanical deformability allows the fastening-type and compression-based shock absorbers to retain the increased mean value of the fracture acceleration at high acceleration. Specifically, the fastening-type shock absorbers with the nanocomposite and the bare CNTs exhibit 70%, and 53% increased mean values of the fracture acceleration compared to that of the hard stop, respectively. Additionally, the compression-based shock absorbers with the nanocomposite and bare CNTs show 115% and 44% increases in the mean value of the fracture acceleration compared to that of the hard stop, respectively. Regarding the friction-based shock absorbers, the improvement in shock stability due to the Al_2_O_3_ layer was negligible compared to the spring stop. Additionally, introducing Al_2_O_3_ does not lead to an improvement in the survival rate of the shock absorber with CNT arrays. This result is attributed to the low surface energy of the Al_2_O_3_ layer^28,32^, which leads to a low adhesion force of the Al_2_O_3_-coated composites, unlike that of the bare CNT array, decreasing the frictional damping at the contact interfaces. Therefore, the survival rate curves show that the friction-based absorbers with the composite indicate lower durability to the shock than those with the bare CNTs due to the decrease in the dissipated shock energy, as shown in Fig. 3e. The friction-based shock absorber demonstrated the lowest shock reliability among the nanocomposite-based absorbers but still exhibited a higher survival rate than that of the control devices. The relatively low survival rate of the friction-based absorber indicates that Coulomb damping generated from frictional contact effectively dissipated the impact energy, but the movable displacement of the proof mass was larger than that of other devices. Thus, it appears that the stress applied to the beam deformation exceeded the critical value of the yield stress of the beam.

Furthermore, the low adhesion force of the individual composite strand prevents permanent deformation and buckling in the nanocomposite arrays. In the bare CNT arrays, when the mechanical shock was applied, permanent deformation occurred due to the high adhesion force between individual strands. In contrast, the low adhesion of the Al_2_O_3_ layer enables the restoration of its original state even after excessive deformation by providing mechanical resilience to the nanocomposite array, which is consistent with the results of previous studies^28^. Therefore, the resilient and deformable nanocomposite allows the fastening-type and compression-based shock absorbers to have an increased survival rate at high acceleration, as shown in Fig. 3f, g. The fastening-type and compression-based shock absorbers with the nanocomposite show 11% and 49% increased acceleration values compared to those based on bare CNT arrays without the Al_2_O_3_ coating, respectively. Among these samples, the compression-based shock absorbers with the nanocomposite show the highest durability compared to the other specimens. It appears that the designed microstructure of the shock absorber induced compressive buckling of the nanomaterials during the collision. Therein, the buckled nanocomposite could be extremely deformed to provide a larger absorbing capacity for mechanical shock.

We sought to estimate the degree of energy absorption and compare it to previous literature. The calculated mass density of each Al_2_O_3_–CNT strand is 2.171 × 10^−15^ g/m^3^, based on a case similar to our synthesis method and considering that the mass densities of Al_2_O_3_ and the multiwall CNT are 3.95 and 1.74 g/cm^3^, respectively^33^. Additionally, we assume that the height of the nanotube bundles is 2.5 μm, determined by the designated gap, and the estimated number density of nanotube arrays is 10^10^–10^11^ cm^−2^ ^34,35^. Moreover, the measured diameter of the CNTs is 10 nm, the targeted coating thickness of the Al_2_O_3_ layer is 2 nm, and the designated size of the Al_2_O_3_–CNT-integrated area is 200 × 50 μm^2^. Based on the previous reports^28^ of the volume-normalized energy absorption amount of the aligned Al_2_O_3_–CNT and the bar CNT compression, we estimate the energy absorption density of Al_2_O_3_–CNTs and bare CNTs to be approximately from 2.5 × 10^6^ J/m^3^ to 3.0 × 10^7^ J/m^3^ and from 10^5^ J/m^3^ to 1.5 × 10^6^ J/m^3^, respectively.

Furthermore, we investigated the fracture acceleration values of three shock absorbers without CNTs and Al_2_O_3_, consisting of friction-, fastening-, and compression-based structures. Figure S8 shows the mean values and standard errors in the fracture accelerations of the specimens without CNTs and Al_2_O_3_. The interdigitated spring stops consist of friction-based and fastening-type absorbers without CNTs and Al_2_O_3_. The mean fracture acceleration values of the interdigitated spring stop and the compression-based stops are 6369 and 6178*g*, respectively. Meanwhile, in the case of using bare CNTs, the friction-, fastening-, and compression-based shock absorbers have mean fracture values of 6427, 7070, and 6649*g*, respectively. The mean values of the specimens without CNTs and Al_2_O_3_ indicate that the designed shock absorbers without CNTs and Al_2_O_3_ have higher survival rates in the mechanical shocks than those of the hard stop and the spring stop. Meanwhile, they have low mean values compared to those with CNTs or Al_2_O_3_–CNTs. Therefore, they could be used as newly advanced designs of spring stops, similar to the cascade beam and self-adaptive nonlinear stops^17,18^, respectively.

Furthermore, we investigated the size of the contact area in the shock absorbers based on friction and compression. However, the size of the contact area did not impact the survival performance. The dominant parameters affecting the survival rate are the structural design of the shock absorber and the usage of Al_2_O_3_–CNTs. As shown in Fig. S9, the size of the contact area did not lead to an increase in the average fracture values, but coating Al_2_O_3_ on the CNT arrays increased the average fracture values of the specimens in the same design. Furthermore, we compare the characteristics of our shock absorbers with other works, as listed in Table S3. Table S3 exhibits the other structural designs, the introduced materials, and the enhanced survival rate compared with the control device.

### Investigation of failed shock absorbers with nanocomposites

**Fig. 4:**
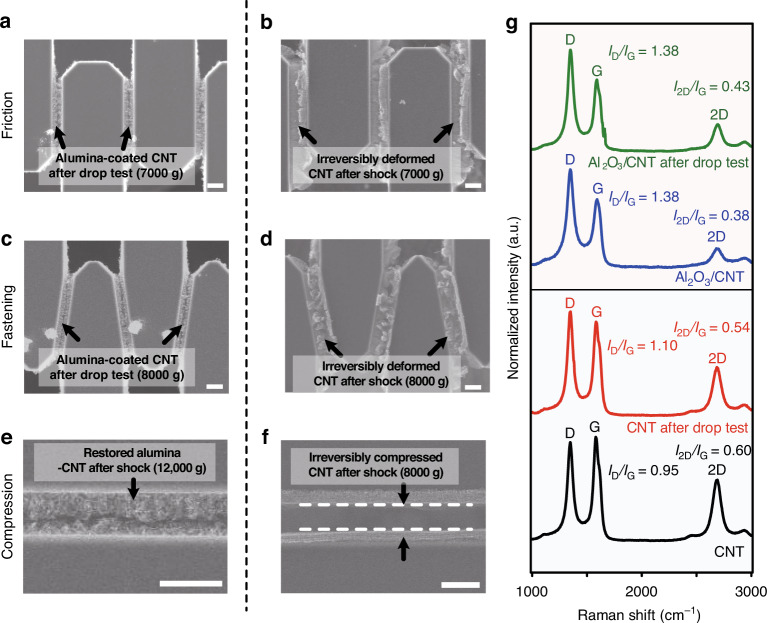
Investigation of the nanocomposite of the fractured specimens after the shock test. SEM images of the shock absorbers with **a**, **b** friction-based, **c**, **d** fastening-type, and **e**, **f** compression-based nanocomposites and CNTs after applying excessive acceleration. The scale bar is 10 μm. **g** Raman spectra of the composite and CNT arrays to investigate the defect changes in the composite and CNT arrays before and after applying the mechanical shock

We also investigated the structural changes in the nanocomposite after excessive loading acceleration. As shown in the SEM images in Fig. 4a, there is no change in the morphology of the nanocomposites, indicating that the friction-based absorber with the nanocomposite has excellent frictional resilience and repeatability. In contrast, the bare CNT arrays were bent after the acceleration was loaded, and the compliant spring did not recover its original position due to the high adhesion force of the bare CNT arrays, as shown in the SEM images in Fig. 4b. The SEM images in Fig. 4c, d shows the nanocomposite and bare CNT arrays in the fastening-type shock absorber after applying the mechanical shock. Due to the simultaneous loading of excessive shear and compressive forces, the bare CNT arrays were entangled and pressed, while the nanocomposite showed the original state with linear interfaces without structural change.

In the compression-based shock absorber, the difference in mechanical resilience of the composites with and without alumina coating is more evident. The SEM images in Fig. 4e, f shows the morphology change in the nanocomposite and the bare CNT arrays, respectively, when a compressive load was applied. As shown in Fig. 4e, there is no change in the aligned structure of the nanocomposite, indicating that the Al_2_O_3_-reinforced CNT nearly recovered to the original state. However, in the device with the bare CNT arrays, the impact force created a gap between the CNT arrays. This indicates that the CNT arrays integrated with the device remained compressed, showing the irreversible character of aligned CNT arrays to compressive stress, which is comparable to that of the previously published literature^31^. This led to subsequent shock exposure without the participation of CNT arrays in the compression-based specimen. Meanwhile, in the case of the fastening devices with bare CNTs, the interdigitated structures were locked by the deformed CNT structures, as shown in Fig. 4d. Thus, the CNTs were involved in the contact as a cushioning layer, leading to a large mean value compared with that of compression-based devices.

We also measured the Raman spectra of the composite and CNT arrays to investigate the defect changes in the composite and CNT arrays after applying mechanical shock. Figure 4g shows the Raman spectra of the composite and CNT arrays before and after the drop test at an acceleration of 12,000*g*. The plotted spectra were normalized to the intensity of the D peak. Generally, the *I*_G_/*I*_D_ ratio and the *I*_2D_/*I*_G_ ratios indicate the degree of defects in carbon bonds and the quality of the *sp*^2^-hybridized carbon arrangement that appears only in CNT- and graphene-based materials, respectively^36^. These ratios decreased after applying the mechanical shock, indicating that the defect and quality of the *sp*^2^ carbon arrangement decreased simultaneously^37^. Meanwhile, there was no noticeable change in the nanocomposite, indicating the mechanical stability of the composite.

## Conclusion

We fabricated a geometrically aligned region-selectively integrated nanocomposite as an in-plane shock absorber for MEMS devices. The aligned CNT arrays exhibit a high aspect ratio, and the reinforcing alumina exhibits low surface energy to provide deformability and mechanical resilience, respectively, as a composite material. These unique nanocomposite characteristics enable a reduction in the acceleration and dissipation of mechanical energy induced by mechanical shock, greatly enhancing the shock reliability of microstructures. As a result, the nanocomposite-based shock absorber exhibited a high survival rate over a wide acceleration range compared with a hard stop, a spring stop, and a CNT-based shock absorber. Additionally, our newly introduced region-selective integration method of nanocomposite fabrication uses a batch process, which is amenable to mass production. With these enhanced shock reliability and mass-producible manufacturing methods, the proposed nanocomposite-based shock absorber can provide new opportunities to develop highly reliable MEMS devices for use in harsh environments and industries with exposure to large shocks.

## Experimental section

### Fabrication of Si microstructures

A Si device layer and a handle layer, whose thicknesses are 50 and 500 µm, respectively, were patterned on a 4-in. silicon-on-insulator wafer by using deep reactive ion etching with photoresist masks. The patterned wafer was then divided into 9 mm × 9 mm scale chips. The buried oxide layer with a thickness of 2 µm was etched using a hydrofluoric acid solution (50% concentration) to release the microstructure.

### Synthesis of vertically aligned CNT arrays and alumina deposition

An iron (Fe) catalyst with a thickness of 2.8 nm for CNT growth was deposited by sputtering using a co-fabricated shadow mask. The undesired Fe catalyst on the top surface was removed by reactive ion etching with carbon tetrafluoride (CF_4_) at 40 sccm, Ar at 300 sccm, trifluoromethane (CHF_3_) at 10 sccm, and N_2_ at 25 sccm under a power of 200 W for 90 s. After the CNT arrays were directly synthesized onto the sidewall surfaces via chemical vapor deposition, Al_2_O_3_ was uniformly coated on all sides of the CNT using ALD (MEMS—ALD, CN-1). The ALD chamber temperature was maintained at 450 °C with purging N_2_. Trimethylaluminum and ozone were used as the metalorganic and oxidizing precursors of Al_2_O_3_, respectively. Al_2_O_3_ with a thickness of 0.8 Å was deposited in each cycle, and the number of cycles was adjusted to achieve the desired thickness.

### Characterization of the microstructures and alumina-reinforced CNT array

The morphologies and Raman spectra of the microstructure and the nanocomposite were observed using a field emission scanning electron microscope (IT-500HR, JEOL) and a Raman spectrometer with an argon-ion layer of 532 nm wavelength (LabRam Aramis, Horiba Jovin Yvon). TEM images were obtained using a high-resolution transmission electron microscope (JEM-F200, JEOL). The value and transient response of the acceleration induced by the drop-collision test were monitored using a high-*g* accelerometer (350D02, PCB Piezotronics) and an oscilloscope (DSO5014A, Agilent). During the shock-survival test, the experiment was conducted in the impact range of 1000–12,000*g* in increments of approximately 200*g*, in which the total number of shocks was 50.

## Supplementary information

Supplemental Material
