# Supplementary material for: Mechanically resilient, alumina-reinforced carbon nanotube arrays for in-plane shock absorption in micromechanical devices
Source: Microsyst Nanoeng. 2023 Jun 8;9:76. doi: 10.1038/s41378-023-00539-7 (PMC10250468; doi:10.1038/s41378-023-00539-7)
Supplement: Supplementary file 1 — Supplemental Material [file 41378_2023_539_MOESM1_ESM.docx]

**Mechanically Resilient, Alumina-Reinforced Carbon Nanotube Arrays for In-Plane Shock Absorption in Micromechanical Devices**

Eunhwan Jo^1, ‡^, Hojoon Lee^1, ‡^,Jae-Ik Lee^2^, and Jongbaeg Kim^1*^

^1^School of Mechanical Engineering, Yonsei University, 50 Yonsei-ro, Seodaemun-gu, Seoul 03722, Republic of Korea

^2^Department of Neurosurgery, Massachusetts General Hospital, Harvard Medical School, 25 Shattuck St, Boston, MA 02115, United States

‡These authors contributed equally.

*Corresponding author: Jongbaeg Kim

E-mail: kimjb@yonsei.ac.kr

**Contents**:

1. Fabrication process of the shock absorbers. (Figure S1)
2. An enlarged scanning electron microscope (SEM) image of Al_2_O_3_–CNTs integrated on the sidewall of the microstructures. (Figure S2)

3. A scanning electron microscope (SEM) image of the shock absorber with bare CNTs. (Figure S3)

4. SEM images indicating design parameters and dimensions of the shock absorbers. (Figure S4)
5. Design parameters and dimensions of the shock absorbers. (Table S1)
6. Experimental setup for measuring the acceleration versus the survival rate. (Figure S5)
7. A transient response of the applied acceleration. (Figure S6)
8. Fitted parameter of the Weibull distribution curves. (Table S2)
9. The acceleration value and the fitted curves with a Weibull distribution. (Figure S7)
10. The mean values and standard errors in the survival rate of the specimens without CNTs and Al_2_O_3_. (Figure S8)

11.The mean values of the fracture acceleration and standard errors with respect to the size of the nanotube’s contact area.(Figure S9)

12. Comparison of reported shock absorber performances (Table S3)


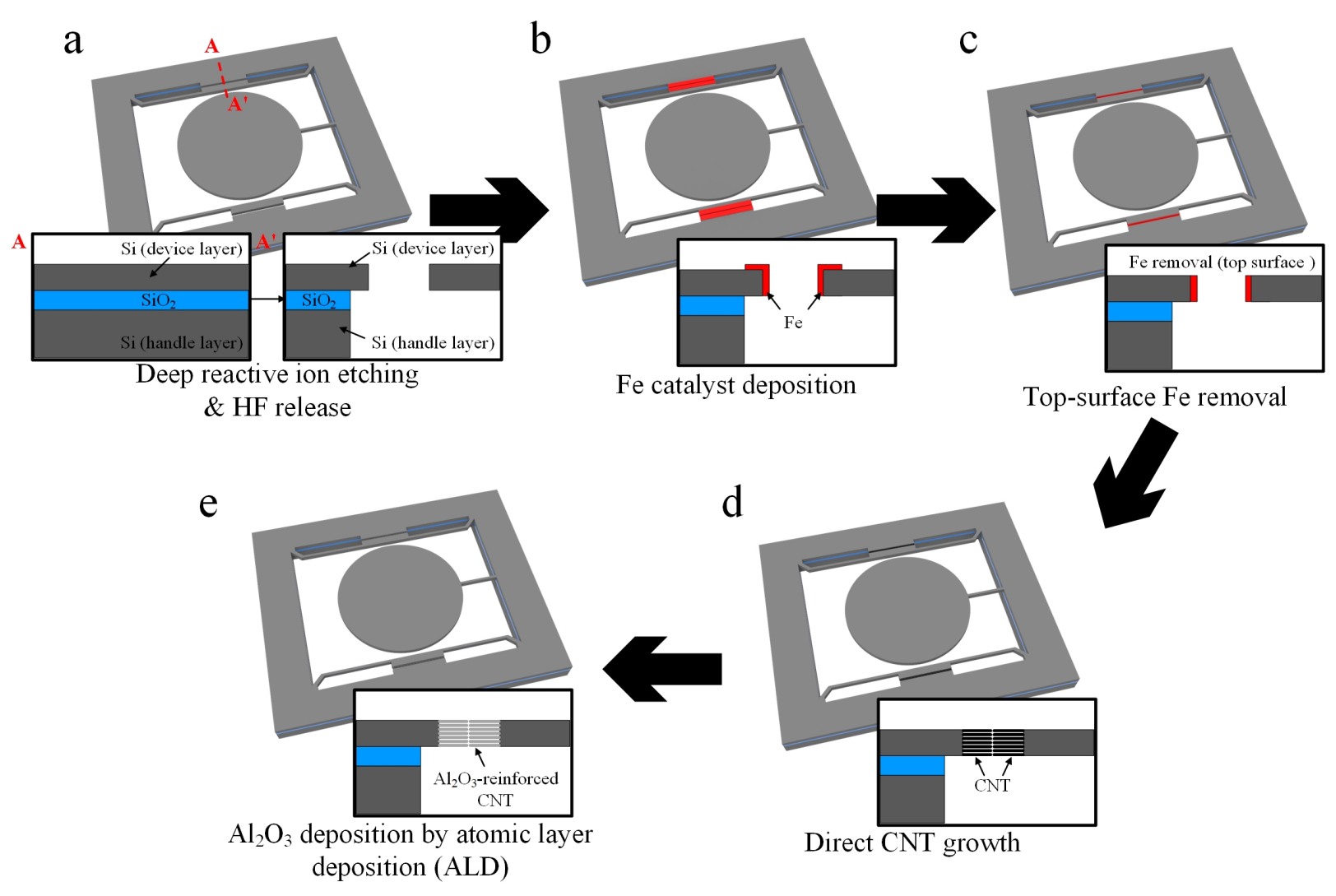


**Fig. S1.** Schematic illustration for a fabrication process of the proposed shock absorber with nanocomposite. **a** A silicon (Si) device layer and a handle layer were patterned on a 4-inch silicon-on-insulator wafer by using deep reactive ion etching with photoresist masks. The patterned wafer was then divided into 9 mm × 9 mm scale chips. The buried oxide layer was etched using a hydrofluoric (HF) acid solution to release the microstructure. **b** An iron (Fe) catalyst with a thickness of 2.8 nm for CNT growth was deposited by sputtering using a co-fabricated shadow mask. **c** The Fe catalyst on the top surface was removed by reactive ion etching. **d** After the CNT arrays were directly synthesized onto the sidewall surfaces via chemical vapor deposition, **e** Al_2_O_3_ was uniformly coated on all sides of the CNT using atomic layer deposition (ALD) equipment.


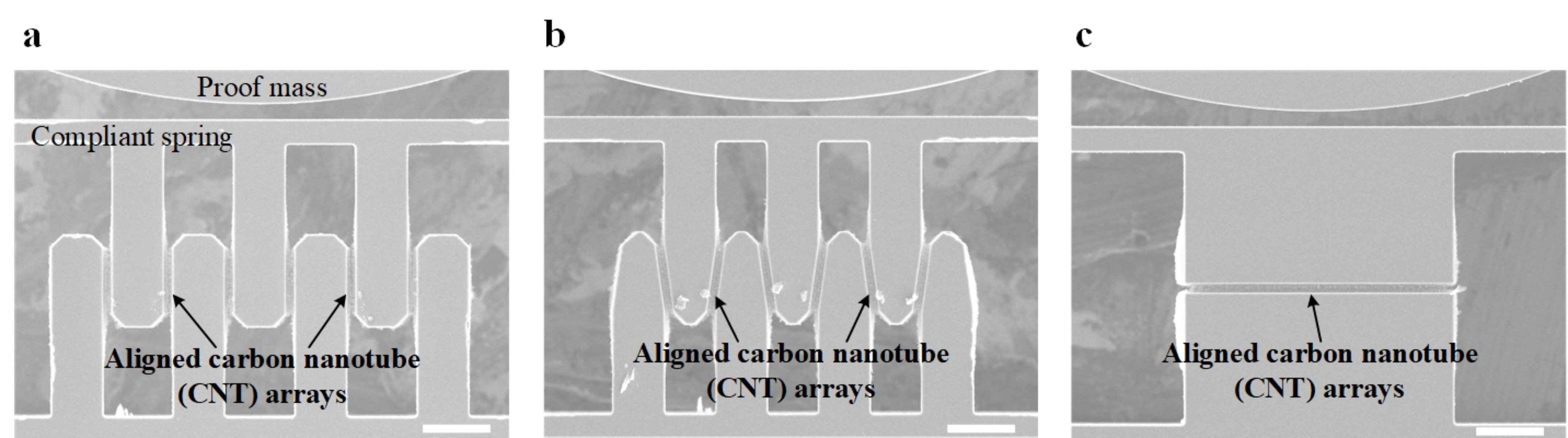


**Fig. S2** Scanning electron microscope (SEM) images of the shock absorbers with the bare CNT arrays synthesized in the designated sidewalls before atomic layer deposition (ALD) of Al_2_O_3_. The scale bar is 50 μm.


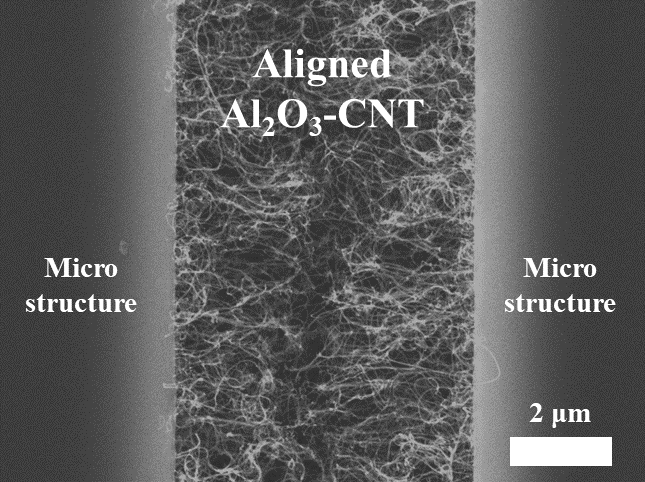


**Fig S3**. Enlarged scanning electron microscope (SEM) image of Al_2_O_3_–CNTs integrated on the sidewall of the microstructures.


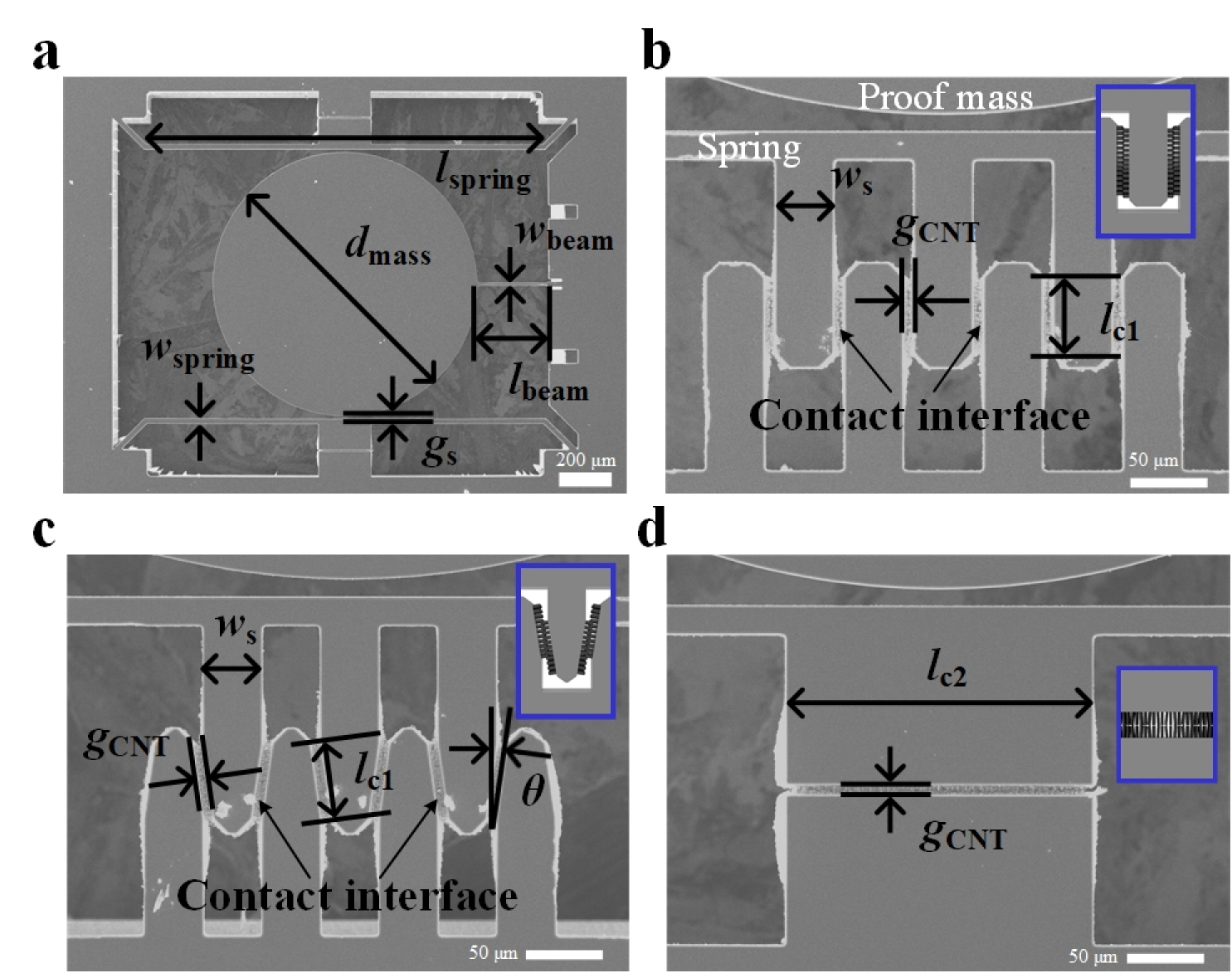


**Fig. S4** Scanning electron microscope (SEM) images indicating **a** the design parameters and dimensions of the tested shock absorbers. **b** The friction-based shock absorber. **c** The fastening-type shock absorber. **d** The compression-based shock absorber.

**Tab. S1** Design parameters and dimensions of the shock absorbers.

| Parameters | Symbols | Dimensions (μm) |
| --- | --- | --- |
| Diameter of the proof mass | *d*_mass_ | 1,000 |
| Length of the compliant spring | *l*_spring_ | 1,500 |
| Width of the compliant spring | *w*_spring_ | 20 |
| Length of the microbeam | *l*_beam_ | 260 |
| Width of the microbeam | *w*_beam_ | 10 |
| Gap between the proof mass and the shock absorber | *g*_s_ | 10 |
| Width of the shock absorber structure | *w*_s_ | 40 |
| Gap in the structures for CNT growth | *g*_CNT_ | 5 |
| Length of the CNT contact interface | *l*_c1_ | 50 |
| Length of the CNT contact interface (compressing type shock absorber) | *l*_c2_ | 100, 200, 300, 400 |
| Taper angle for the fastening structure | *θ* | 2°, 4°, 6°, 8°, 10° |
| Number of contact interfaces on a single shock absorber | *#* | 2, 4, 6 |


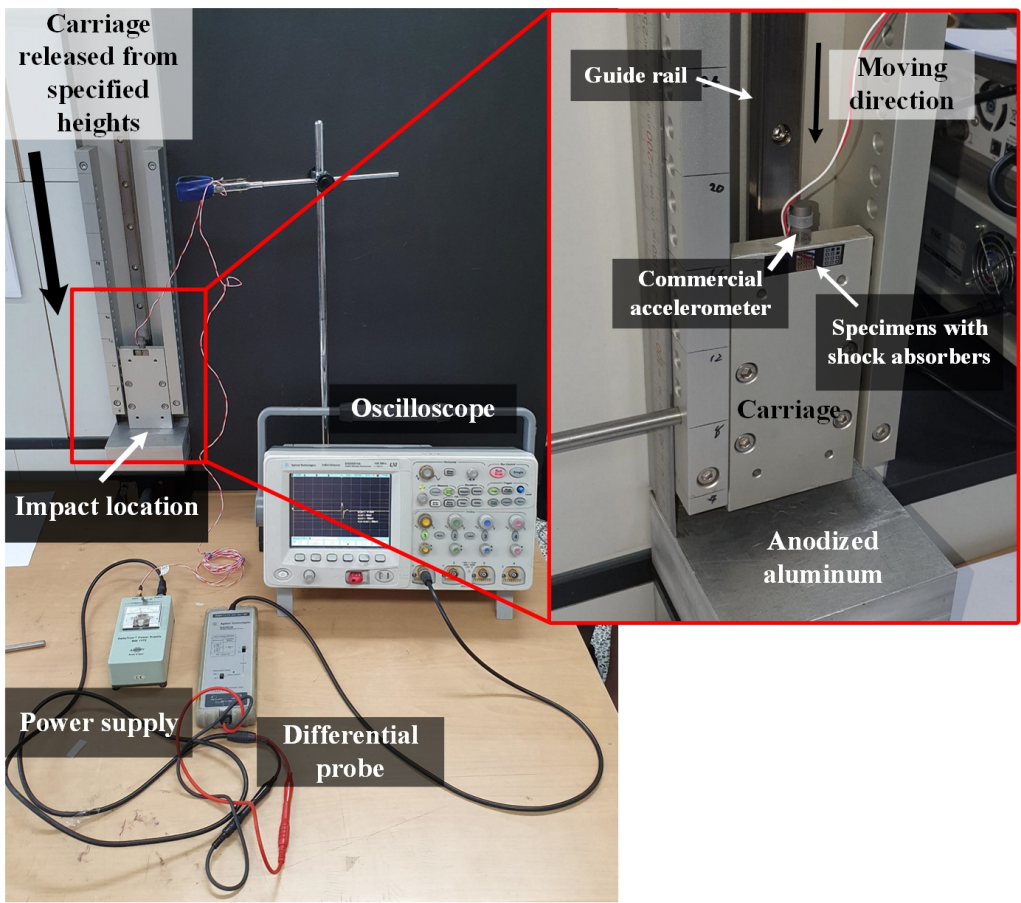


**Fig. S5** Experimental setup for measuring the acceleration versus the survival rate.


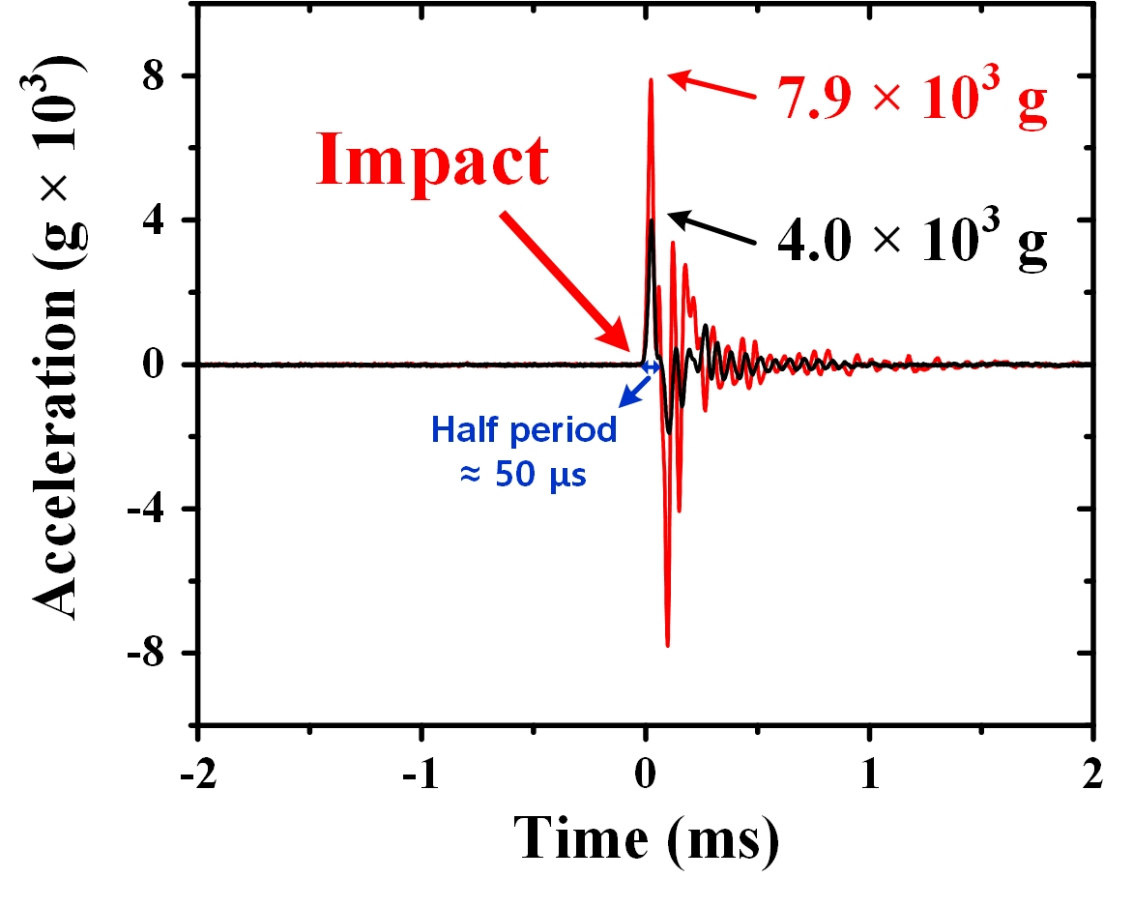


**Fig. S6** The measured transient response of the applied acceleration with a sinusoidal half period of approximately 50 μs.

**Tab. S2** Fitted parameter of the Weibull distribution curves

|  |  | |  | | Alumina-CNT | | | Bare-CNT | | |
| --- | --- | --- | --- | --- | --- | --- | --- | --- | --- | --- |
|  | | Hard stop | | Spring stop | Friction | Fastening | Compression | Friction | Fastening | Compression |
| α | | 5068 | | 5804 | 6171 | 7937 | 10106 | 6526 | 7298 | 7095 |
| β | | 4.909 | | 8.539 | 5.695 | 17.081 | 12.763 | 12.445 | 9.796 | 5.460 |
| *R*^2^ | | 0.8864 | | 0.9215 | 0.9661 | 0.9874 | 0.9663 | 0.9725 | 0.9935 | 0.9655 |

$$y= e^{{-(\frac{x}{\alpha})}^{\beta}}$$

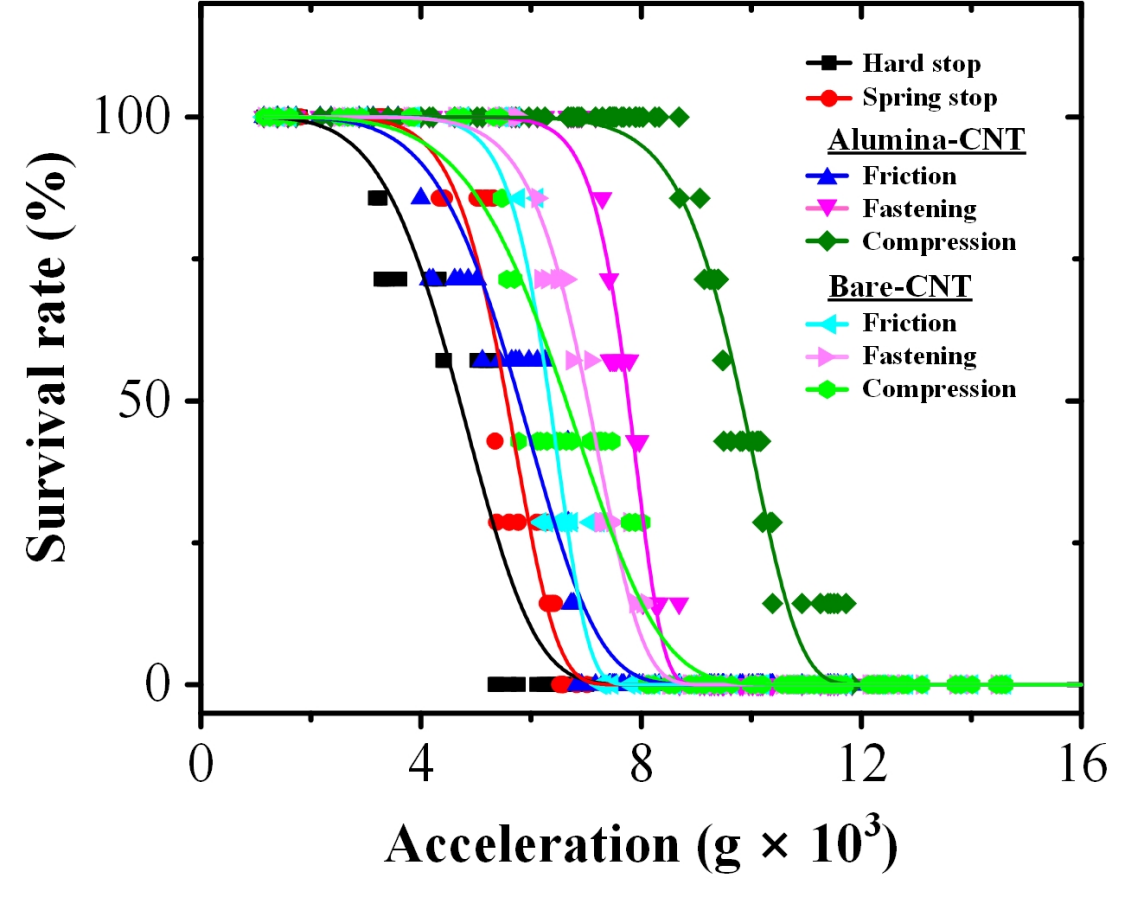


**Fig. S7** The measured acceleration value and the fitted curves with a Weibull distribution to predict the failure rate of the devices as a function of a shock exposure.


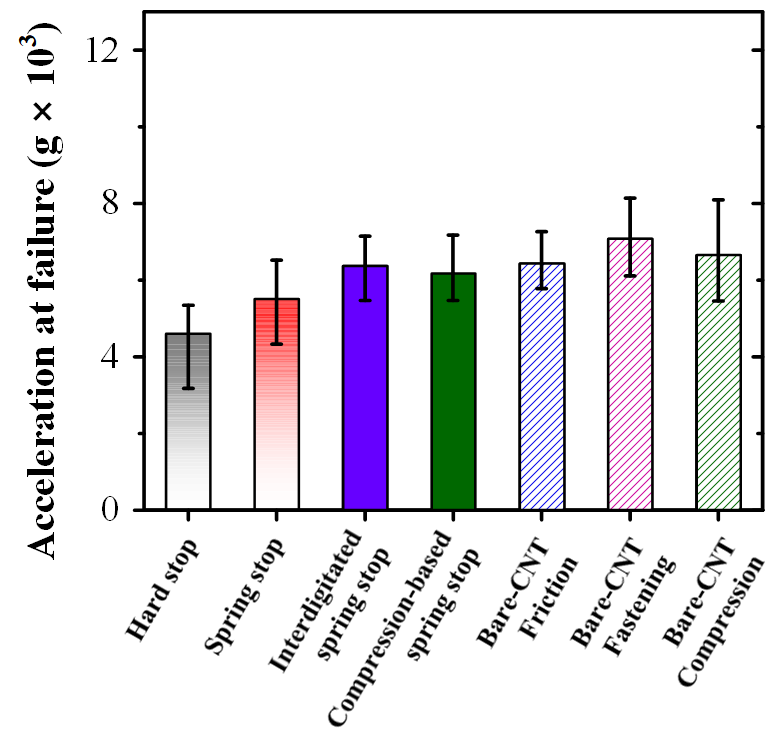


**Fig S8**. The mean values and standard errors in the survival rate of the specimens without CNTs and Al_2_O_3_. These mean values indicate that the designed shock absorbers without CNTs and Al_2_O_3_ is higher survival rate in the mechanical shocks than those of the hard stop and the spring stop.


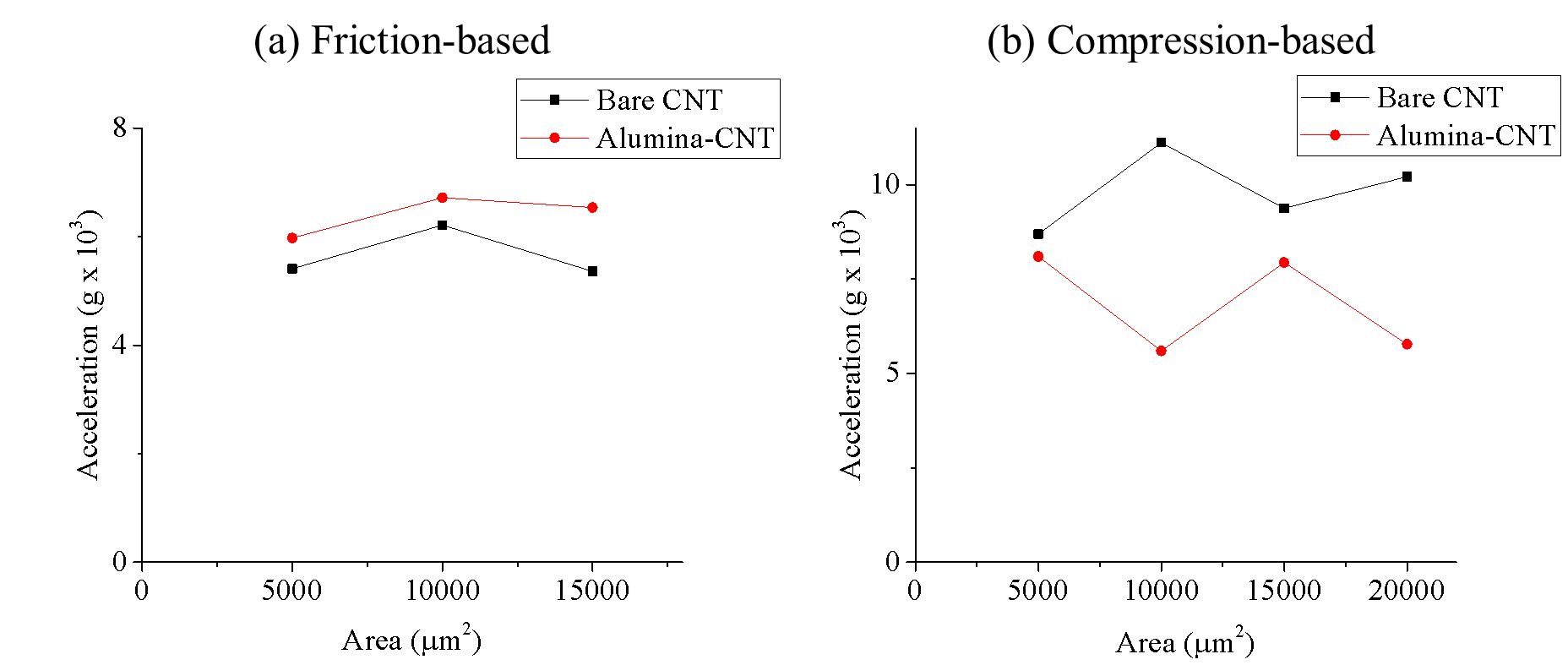


**Fig S9**. The mean values of the fracture acceleration and standard errors of (a) friction-based specimens and (b) compression-based specimens with respect to the size of the nanotube’s contact area.

**Table S3.** Comparison of reported shock absorber performances

| Material | Structural design | Average acceleration at failure | | | α (acceleration  at 36.79%  survival rate) | | Enhanced rate  (compared with the control device) | | Reference |
| --- | --- | --- | --- | --- | --- | --- | --- | --- | --- |
|  |  | Shock absorber | | Control device | Shock absorber | Control device |  |  |  |
| Si | Nonlinear spring |  |  | |  |  | N/A | | 14 |
| Parylene | Soft thin film |  |  | |  |  | N/A | | 14 |
| Si | Latching stop |  |  | | 5460 | 2580 | 112 % | | 16 |
| Si | Latching stop |  |  | | 5149 | 2580 | 100 % | | 17 |
| Sn_3.0_Ag_0.5_Cu | Ductile solder ball |  |  | | 6419 | 3285 | 95 % | | 20 |
| Alumina-coated CNT | Friction-based | 5739 | 4607 | | 6171 | 5068 | 25 % | 22 % | This work |
| Alumina-coated CNT | Fastening-type | 7828 | 4607 | | 7937 | 5068 | 70 % | 57 % | This work |
| Alumina-coated CNT | Compression-based | 9893 | 4607 | | 10107 | 5068 | 115 % | 99 % | This work |
